# Supplementary material for: Developmental Profile of Sleep and Its Potential Impact on Daytime Functioning from Childhood to Adulthood in Sickle Cell Anaemia
Source: Brain Sci. 2020 Dec 14;10(12):981. doi: 10.3390/brainsci10120981 (PMC7764980; doi:10.3390/brainsci10120981)
Supplement: Supplementary file 1 [file brainsci-10-00981-s001.zip › Supplementary_Materials/S1.pdf]

Supplementary Table 1

Adapted version of the Children's Sleep Habits Questionnaire (CSHQ) for the use in sickle cell disease

Current Nighttime Symptoms	Never	Not often (<1 night week)	Sometimes (1-2 nights week)	Often (3-5 nights week)	Always (6-7 night/s week)	Dont know
Snores						
Difficulty breathing while asleep						
Stops breathing during sleep						
Noisy breathing						
Restless sleep						
Sweating when sleeping						
Nightmares						
Sleep walking						
Sleep talking						
Screaming in his/her sleep						
Grinds his/her teeth						
Wets the bed						
Resists going to bed at bedtime						
Trouble falling asleep						
Feels like s/he can't move arms or legs when falling asleep						
Wakes up at night						
Gets out of bed at night						
Trouble staying in his/her bed at night						
Kicks or jerks legs in sleep						
Uncomfortable feelings in his/her legs; creepy/crawly before falling asleep						

Sleep Disordered Breathing
0 - 16 points

Parasomnias
0 - 32 points

Bedtime Resistance
0 - 4 points

Night Wake
0 - 12 points

Movement at night
0 - 8 points

Higher scores = more sleep related problems

CSHQ Composite Score

No sleep problems 0 points — Sleep problems 80 points
